# Defective NKT Cell Activation by CD1d+ TRAMP Prostate Tumor Cells Is Corrected by Interleukin-12 with alpha-Galactosylceramide

**DOI:** 10.1371/journal.pone.0011311

**Published:** 2010-06-25

**Authors:** Michael Nowak, Mohammed S. Arredouani, Adrian Tun-Kyi, Ingo Schmidt-Wolf, Martin G. Sanda, Steven P. Balk, Mark A. Exley

**Affiliations:** 1 Division of Hematology/Oncology, Department of Medicine, Beth Israel Deaconess Medical Center, Harvard Medical School, Boston, Massachusetts, United States of America; 2 Department of Hematology and Oncology, University of Bonn, Bonn, Germany; 3 Division of Urology, Department of Surgery, Beth Israel Deaconess Medical Center, Harvard Medical School, Boston, Massachusetts, United States of America; New York University, United States of America

## Abstract

Numerical and functional defects of invariant natural killer T cells (iNKT) have been documented in human and mouse cancers, resulting in a defect in IFN production in several malignancies. iNKT cells recognize glycolipids presented on CD1d molecules by dendritic and related cells, leading to their activation and thereby regulating immune reactions. Activated iNKT cells cytokine secretion and cytotoxicity can inhibit existing and spontaneous tumor growth, progression, and metastasis. We have identified functional iNKT cell defects in the murine TRAMP prostate cancer model. We found that iNKT cells show the ability to migrate into TRAMP prostate tumors. This infiltration was mediated through CCL2: CCR5 chemokine: receptor interaction. Prostate tumor cells expressing CD1d partially activated iNKT cells, as appreciated by up-regulation of CD25, PD-1 and the IL-12R. However, despite inducing up-regulation of these activation markers and, hence, delivering positive signals, prostate tumor cells inhibited the IL-12-induced STAT4 phosphorylation in a cell-cell contact dependent but CD1d-independent manner. Consequently, tumor cells did not induce secretion of IFNγ by iNKT cells. Blocking the inhibitory Ly49 receptor on iNKT cells in the presence of α-GalCer restored their IFNγ production in vivo and in vitro. However, Ly49 blockade alone was not sufficient. Importantly, this defect could be also be reversed into vigorous secretion of IFNγ by the addition of both IL-12 and the exogenous CD1d ligand alpha-galactosylceramide, but not by IL-12 alone, both in vivo and in vitro. These data underscore the potential to optimize iNKT-based therapeutic approaches.

## Introduction

Invariant natural killer T (iNKT) cells are a subset of lymphocytes with an important role in regulating immune responses, including immune surveillance. iNKT cells recognize lipid antigens presented by the monomorphic MHC-like molecule CD1d. They were initially identified based on their restricted T cell receptor repertoire used. The hallmark of iNKT cells and basis of their regulatory function is the rapid secretion of multiple cytokines upon TCR triggering accompanied with an increased cytotoxic capacity [1]. The cytokines secreted by iNKT cells include both regulatory cytokines (e.g. IL-4, IL-10) as well as pro-inflammatory cytokines such as IL-2, and IFNγ, reflecting their capacity to suppress or stimulate immune responses [2]. Although a panel of exogenous CD1d ligands including the prototypic high-affinity lipid alpha-galactosylceramide (α-GalCer) have been reported to date, the identity of physiological endogenous ligands that mediate CD1d-dependent iNKT cell activation remains ambiguous [3]. While the function of iNKT cells as positive or negative regulators of immune responses has been widely acknowledged [2], [4], the exact mechanisms polarizing their effector functions are elusive.

Studies in multiple human cancers and animal models revealed numerical and functional defects in iNKT cells. Decreased numbers of circulating iNKT cells were accompanied by a decrease in IFNγ production by iNKT cells in advanced prostate cancer and other cancers, concurrent with increased IL-4 production upon re-stimulation *in vitro* [*5*]–[9**]. The activation of iNKT cells with α-GalCer promotes tumor rejection and protected from the development of metastasis in multiple mouse models and injection of α-GalCer-pulsed DC further improves this effect [10]–[12]. However, previous studies also showed that some CD1d-restricted non-invariant NKT cells can suppress anti-tumor responses [13].

The contribution of iNKT cells in immune surveillance is at least partly based on their capacity to mature dendritic cells (DC) and subsequently activate natural killer (NK) cells, the latter of which become potent cytotoxic cells. Upon recognition of CD1d:lipid complexes and the co-stimulatory molecules CD80/86 on the surface of DCs, iNKT cells up-regulate the IL-12R and CD40L molecules. Subsequently and mediated by CD40L iNKT cells then induce maturation and production of IL-12 in DCs. This IL-12 release potently increases IFNγ production by iNKT cells [14], [15].

IL-12 was initially described as a master regulator of Th1 responses [16], and, like α-GalCer, drives the anti-metastatic activity of T cells including iNKT cells [10]. Several pre-clinical and clinical trials using IL-12 have been conducted to improve immunotherapy for cancers [17]. Whether defects of iNKT cells are mediated by intrinsic causes or by an impaired stimulatory capacity of DC present in the tumor microenvironment is not known. Moreover, it is not known whether DC and iNKT cells undergo a sustained functional inhibition in tumors or whether their functions can be restored by adjuvants like IL-12 *in vivo*.

In this study we sought to establish a murine prostate cancer model to study iNKT cell defects and determine if their functions are inhibited by tumor cells. We found functional iNKT cell defects in the prostate cancer model TRAMP (transgenic adenomacarcinoma of the mouse prostate) resembling those functional iNKT cell defects seen in human malignancies. iNKT cells were attracted by tumor cells to migrate into prostate tumors mediated through the CCL2-CCR5 axis. Both primary prostate tumors as well as mouse and human CaP cell lines express high levels of CD1d, permitting direct interaction with iNKT cells.

We found that prostate tumor cells induced the production of Th2 cytokines by iNKT cells. These tumor cells and murine prostate tumors specifically induced the up-regulation of activation markers IL-12R, CD25 and PD-1 on iNKT cells. Parallel, TRAMP-C2 cells cell-contact dependently blocked IL-12 mediated STAT4 phosphorylation in iNKT cells. This aberrant iNKT cell activation was correctable by simultaneous addition of the exogenous CD1d ligand α-GalCer and IL-12, and allowed iNKT cells to produce IFNγ in response to tumor cells. Blocking Ly49 inhibitory NK receptor also rescued the IFNγ production of iNKT cells stimulated by TRAMP-C2 cells in the presence of α-GalCer. Conceivably, the restoration of the iNKT cell functions (e.g. IFNγ production) by addition of IL-12 or Ly49 neutralization provides a tempting possibility for overcoming iNKT cell defects in malignancy.

## Materials and Methods

### Mice

C57Bl/6J were purchased from Jackson Laboratories (Bar Harbor, MA). TRAMP mice were described previously [18]. For experiments, 6- to 8-wk-old and 15- to 35-wk-old C57BL/6J mice and heterozygous TRAMP mice at 15 to 35 weeks of age were used. All mice were housed in the specific pathogen-free animal facility at Beth Israel Deaconess Medical Center. Animal experiments were approved by the Institutional Animal Care and Use Committee (protocol 075-2009).

### Reagents

PBS57-loaded and empty CD1d monomers and tetramers were provided by the NIH tetramer facility (Emory Vaccine Center, Atlanta, GA). The following monoclonal antibodies (mAbs) and secondary reagents were used: mouse CD1d PE (1B1), human CD1d PE (42.1), CD3 FITC, CD4-APC-Cy7, NK1.1 PE, purified anti-CD16/32, CD69 PE-Cy7, CD212 biotin, purified anti-CD16/32, streptavidin-APC-Cy7, were from BD Biosciences. αβTCR AlexaFluor700, PD-1 PE, CD25 PerCP-Cy5.5, I-A/I-E Pacific Blue, purified anti-Ly49C/F/H/I were purchased from eBioscience, anti-PD-L1 and Ly49C/F/I/H were purchased from Biolegend and pSTAT4 (Ser721) was purchased from Santa Cruz.

### Cell culture of TRAMP-C2

Cells were cultured in RPMI-1640 supplemented with Penicillin and Streptomycin (Mediatech, Manassas, VA) and 5% FCS (Hyclone, Logan, UT). The cell lines TRAMP-C2, DU145, PC3, LNCaP and Hela were maintained in cell culture medium and split using trypsin digestion when 80% confluence was reached. The prostate epithelia PrEC were maintained in PrEGM Bullet Kit medium (Cambrex, Charles City, IA) and split every 2–4 days by brief trypsination.

### Bone-marrow derived DC (BM-DC)

BM-DC were generated from wildtype (WT) mouse BM in the presence of GM-CSF as described by Inaba et al. [19] with modifications. Briefly, BM cells were cultured in complete medium supplemented with 5% culture supernatants of GMCSF-producing B16F10 (kind gift of Dr. Dranoff, Dana-Farber Cancer Institute, Boston, MA), replaced every other day until day 6 of culture.

### Spleen and liver preparations

Single cell suspensions from spleens were prepared by standard techniques. Liver mononuclear cells (MNC) were isolated as previously described [20] without prior Collagenase digestion. Briefly, livers were perfused with PBS, minced and MNC were enriched by centrifugation in a two-step (40%/ 60% (w/v) Percoll) gradient. Enriched populations typically contained 20–30% iNKT cells.

### Tumor-infiltrating lymphocytes (TIL)

Primary tumors of TRAMP mice were minced and digested for 1 hr at 37°C in complete medium with Collagenase (Stemcell Technologies, Vancouver, BC, Canada), Hyaluronidase (10 U/mL) and DNase I (10 µg/ml, Sigma-Aldrich, St.Louis, MO). TIL were further enriched by Percoll centrifugation as described [20].

### In vivo stimulation of iNKT cells

TRAMP or C57BL/6 mice were injected i.p. with 2 µg α-GalCer or vehicle (Tween-20). Blood was collected from sacrificed mice by heart puncture at indicated timepoints and coagulated at RT. Sera were collected after centrifugation at 400×g for 10 min and stored at −80°C until further analysis.

### In vivo stimulation of iNKT cells

C57BL/6 mice were injected with 5×10^6^ exponentially grown TRAMP-C2 cells in PBS s.c. in both flanks. Two weeks later mice were i.p. injected with combinations of 150 µg Brefeldin A (Sigma-Aldrich), 1 µg α-GalCer, and 500 ng recombinant mouse IL-12 (RndSystems). 5 hours later livers and spleen were taken and immediately stored in ice-cold PBS.

### Ex vivo stimulation of iNKT cells

iNKT cells from livers were stimulated in the presence of either BM-DC or TRAMP-C2 cells (pulsed with α-GalCer or vehicle). BM-DC or TRAMP-C2 cells were pulsed with α-GalCer (200 ng/ml) for 3 hrs at 37°C and washed three times with medium.

### ELISA

Cytokine-specific ELISA assays (eBioscience, San Diego, CA) were performed following the manufacturers instructions. Sera were diluted 1:10 in PBS/1% BSA.

### Immunofluorescence and microscopy

TRAMP-C2 cells were cultured on cover slips overnight and fixed using methanol/ aceton. Nonspecific binding was reduced with 1 µg/ml normal rat serum in PBS/1% BSA and stained with DAPI and PE-conjugated anti-CD1d mAbs (BD Biosciences, San Jose, CA) at 4°C. Imaging was performed using a Axiovert microscope (Zeiss, Jena, Germany).

### Flow cytometry

Subsequently to blocking with anti-CD16/32 mAbs cells were stained with indicated mAbs and CD1d tetramers. Flow cytometry was performed on a FACS LSR II (BD Biosciences, San Jose, CA). Flow cytometry data were analyzed using FlowJo 8 (Treestar, Ashland, OR).

### Intracellular cytokine stainings

Spleen cells and liver MNC were blocked with anti-CD16/32 mAbs, washed and stained using mAbs against αβTCR, NK1.1. After washing cells were fixed for 15 mins. with 2% Paraformaldehyde in PBS. Cells were then permeabilized with 0.5% Saponin, 1% BSA in PBS for 15 mins. All further steps were carried out in permeabilization buffer. Cells were washed and stained with mAbs against IFNγ, IL-4 or rat IgG1 isotype controls before two final washes with permeabilization buffer and PBS, respectively.

### Western Blot

Cell lysates were separated by SDS-PAGE (12.5%) under reducing or non-reducing conditions as shown and subsequently transferred to PVDF membranes, which were blocked with 5% milk in TBS-T (TBS, pH 8.0, 0.1% Tween 20). The membranes were then incubated with rat anti-mouse CD1d IgM (3C11) antibody (1:1000 dilution) or rat anti-mouse CD1d IgG HB323 (1 µg/ml; kind gift of A. Bendelac) overnight at 4°C. Subsequently, the membranes were washed and incubated with a secondary HRP conjugated goat anti-rat IgM or anti-IgG antibody at 1:2000 (Pierce). Bands were visualized using enhanced chemiluminescence (ECL, Amersham Biosciences).

### Migration assay

Migration assays were performed in 24-well plates with 5 µm transwell inserts (Corning Costar). Liver MNC were pre-incubated with anti-CCR5 or isotype control mAbs (each 50 µg/ml) 15 min before adding to transwell inserts (2.5×10^4^/well) and allowed for 3 hrs to migrate against TRAMP-C2 cells. Migrated non-adherent cells were stained with PBS57-loaded CD1d tetramers and mAbs against CD3, NK1.1, αβTCR. 1×10^4^ splenocytes loaded with Indo-1 (Invitrogen, Carlsbad, CA) were added to samples and analyzed by FACS. Numbers of migrated iNKT cells were calculated using the formula:
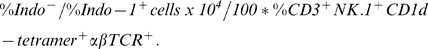



### Statistical analyses

Results are expressed as mean ± SD. For statistical analyses the One-way-ANOVA with Newman-Keuls post-test was used. Values of p<0.05 were considered as significant. Data were analyzed using Prism 5 (GraphPad, La Jolla, CA).

## Results

### Impaired iNKT cell cytokine responses in tumor-bearing mice

We first sought to analyze whether prostate cancer-bearing mice exhibit numerical and functional iNKT cell defects comparable to those reported in cancer patients [6]–[9].

**Figure 1:**
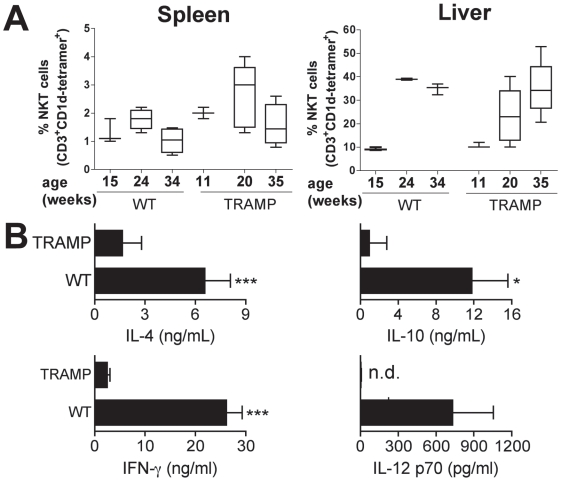
Tumor-bearing TRAMP mice exhibit defective cytokine production in vivo by iNKT cells and DC, albeit harboring normal iNKT cell frequencies. **A.** Cell suspensions of spleens and livers of WT and TRAMP mice were stained with mAbs against CD3, NK1.1 and PBS-57-loaded CD1d tetramers and analyzed by FACS, identifying comparable numbers of iNKT cells. (n = 5). **B.** TRAMP mice or age-matched C57Bl/6 mice were injected with α-GalCer. Mice were sacrificed 90 min (IL-4, IL-10) and 300 min later (IFNγ, IL-12 p70) and cytokines determined by ELISA. (*, p,0.05, ***, p<0.001, n.d, not detectable, n = 3).

We quantitated iNKT cell frequencies from spleens and livers of TRAMP and WT control mice by flow cytometry (Fig. 1A). iNKT cells can be identified by their reactivity to CD1d tetramers loaded with the α-GalCer analogue PBS57 [21]. Consistent with a previous report [22], both groups of mice showed age-dependent increases in liver iNKT cell frequencies. In addition, we observed age- but not strain-dependent increases in absolute iNKT cell numbers in livers and spleens (data not shown). In contrast to humans where relative large cohorts of cancer patients and healthy individuals have been studied, in mice we found that tumor-bearing mice exhibited iNKT cell frequencies comparable to age-matched WT mice (Fig. 1A).

To assess iNKT cell function, WT and TRAMP mice were injected with the exogenous CD1d ligand α-GalCer to activate iNKT cells systemically. We then measured IFNγ as a Th1 cytokine, the Th2 cytokines IL-4 and IL-10, all of which are produced by iNKT cells. Additionally, we measured IL-12, a product of DC. Upon CD1d:TCR interaction in WT mice DC are stimulated by iNKT cells to produce IL-12 which in turn enhances the IFNγ production of iNKT cells. The injection of α -GalCer to WT mice resulted in enhanced serum levels of IL-4 and IL-10 at 90 minutes after activation. At 5 hrs after injection we detected high levels of IFNγ and IL-12 in the serum (Fig. 1B). TRAMP mice, however, showed significantly reduced levels of IL-4, IL-10 and IFNγ upon α-GalCer administration. Concomitantly to the reduced IFNγ levels in TRAMP mice, IL-12 was undetectable upon α-GalCer administration. Collectively, these results show pronounced defects in the cytokine production of both DC and iNKT cells in tumor-bearing mice. This defect involved Th1 as well as Th2 cytokines.

### CCR5-mediated iNKT cell migration into murine prostate tumors

**Figure 2:**
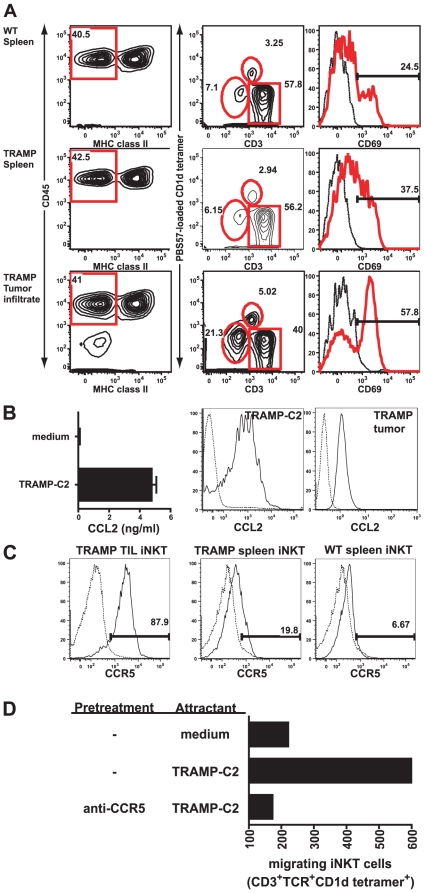
iNKT cells migrate into murine prostate tumors. **A.** WT splenocytes (row 1), TRAMP splenocytes (row 2), and TRAMP prostate-TIL (row 3) were stained with indicated mAbs. CD45^+^MHC class II^−^ cells (left) were gated and analyzed for the expression of CD69. **B.** Left: CCL2 content in culture medium of TRAMP-C2 cells cultured overnight as measured by ELISA. Histograms: CCL2 expression in TRAMP-C2 cells (left) and primary TRAMP tumor cells (right), (dashed lines: isotype controls). **C.** CCR5 expression on gated iNKT cells shown in A (dashed lines: isotype controls). **D.** Liver IHL were incubated with medium, anti-CCR5 or isotype controls and allowed to migrate towards TRAMP-C2 or medium. Migrated iNKT cells were quantified by flow cytometry. Data shown in A–C, and D are representative of 3 and 2 experiments, respectively.

We next questioned whether iNKT cells infiltrate the primary prostate tumors of TRAMP mice. TRAMP tumors were enzymatically dissociated and TILs were enriched by Percoll density gradient centrifugation. To identify iNKT cells, we gated on CD45^+^CD3^+^CD1d tetramer^+^ cells. As Fig. 2A shows, we identified a distinct population of iNKT cells in the lymphocyte infiltrate of TRAMP tumors (mean of 3.65%) comparable to frequencies in the spleens of both groups (Fig. 1A). Based on results shown in Fig. 1 spleens of both groups harbored similar frequencies of iNKT cells. In contrast, we found a larger population of iNKT cells among the lymphocytes in prostate tumors compared to spleens, suggesting iNKT cells are actively recruited into these tumors.

The presence of iNKT cells in the tumor infiltrate prompted us to ask whether the mouse prostate tumor cells attract iNKT cells. Both murine and human iNKT cells express a chemokine receptor repertoire indicative of preferential homing into peripheral non-lymphoid tissues and to sites of inflammation [23], [24]. Human prostate tumors as well as well as primary prostate epithelial cells (PrEC) are known to secrete the chemokine CCL2 (MCP-1) [25], [26]. The expression of CCL2 in mouse tumors and TRAMP mice in particular is not known. CCL2 binds the two chemokine receptors CCR5 and CCR2 which is expressed on iNKT cells [24], [27]. CCL2 has recently been shown to mediate iNKT cell migration into human neuroblastoma [28].

We first tested whether mouse prostate tumors and the derived cell line TRAMP-C2 secrete CCL2. TRAMP-C2 cells produce CCL2 as appreciated by intracellular staining for CCL2 (Fig. 2B, right) and secrete CCL2 (Fig. 2B. bar graph). Importantly, primary tumors of TRAMP mice were found to produce CCL2 as shown by flow cytometry (Fig. 2B, right histogram).

We hypothesized that the relative abundance of iNKT cells among the TIL compared to spleen would be reflected in preferential chemokine receptor expression. In contrast to spleens of both groups the majority of iNKT cells in the prostate tumors were found to express high levels CCR5 on their surface (Fig. 2C). In contrast to CCR5 we did not observe a higher expression of CCR2 on TIL-iNKT cells (data not shown), excluding a critical role of CCR2 in the migration of iNKT cells into tumors.

Employing a transwell migration system, we next asked whether attraction of iNKT cells is mediated by CCR5 (Fig. 2D). We found that TRAMP-C2 cells producing CCL2 stimulated iNKT cell migration through transwell membranes as compared to the medium control. The pre-incubation of liver MNC with blocking anti-CCR5 antibodies reduced the migration of iNKT cells to background levels. Collectively, these data indicate the CCL2:CCR5-mediated iNKT cell attraction by prostate tumor cells and strongly suggest that CCR5 and CCL2 play critical roles in iNKT cell migration into prostate tumors.

### Murine prostate tumors express functional CD1d

CD1d is expressed primarily by hematopoetic cells [29], [30], but low levels of expression also have been found on hepatocytes and intestinal epithelial cells [31], [32]. With the exception of tumors arising from naturally CD1d^+^ tissues and cell types such as lymphoid and myeloid neoplasm [33], [34], the expression pattern of CD1d on malignant cells is largely unknown. The relative abundance of iNKT cells in the TRAMP tumor led us to investigate whether murine prostate cancer cells express CD1d and can activate iNKT cells by CD1d-dependent mechanism.

**Figure 3:**
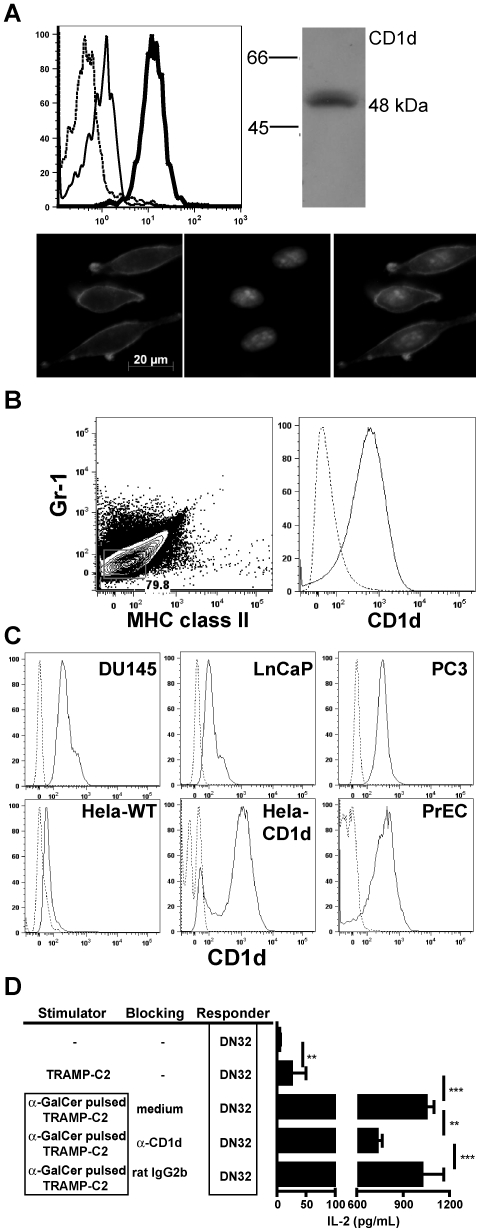
Prostate tumors, primary prostate epithelium, and prostate tumor cell lines express CD1d. **A.** Row 1: CD1d expression of TRAMP-C2 cells (bold line) and WT splenocytes (thin line)(dashed line: isotype control), as analyzed by FACS. Top right: CD1d protein expression of TRAMP-C2 cells as determined by Western Blot using rat-anti-mouse CD1d mAb (3C11). Row 2: Fluorescence micrographs show TRAMP-C2 cells stained against mouse CD1d (left) and DAPI (center). **B.** CD1d expression of primary mouse prostate tumors. Contour plot: Gating of Gr-1^−^, MHC-classII^−^ cells from CD45^−^ tumor cells. Histogram: CD1d expression on gated tumor cells stained against mouse CD1d (solid) or isotype control (dashed line). **C.** CD1d expression in human CaP cell lines and primary prostate epithelium stained with human CD1d or isotype controls (dashed lines). **D.** TRAMP-C2 cells express functional CD1d. TRAMP-C2 were pulsed with α-GalCer or vehicle, washed, loaded with anti-CD1d or isotype control mAbs and incubated with DN32 iNKT hybridoma cells overnight. IL-2 in cell culture supernatants was measured by ELISA. Data shown are representative of 3 experiments (**, P<0.005; ***, p<0.001).

TRAMP-C2 cells stained with CD1d-specific mAbs and analyzed by flow cytometry showed clear CD1d surface expression that was higher than expression on splenocytes used as positive controls. The CD1d expression was further confirmed by Western blot using the rat-anti mouse CD1d IgM clone 3C11 (a similar staining was obtained using the rat anti-mouse CD1d IgG clone HB323, data not shown) and fluorescence microscopy (Fig. 3A). To assess CD1d expression by primary prostate tumor cells from TRAMP mice were isolated by enzymatic dissociation and stained with mAbs against lineage-markers CD45, Gr-1, MHC-II, and CD1d. Gating on lineage marker-negative tumor cells revealed a clear signal of CD1d expression (Fig. 3B).

To determine whether the CD1d expression on these T-antigen induced prostate tumors would translate into human prostate cancers, we tested a panel of human CaP cell lines. DU-145, PC3, and LNCaP expressed CD1d on their surface compared to the CD1d transfected cell line Hela (Fig. 3C). Significantly, also the untransformed human prostate epithelial cells (PrEC) expressed CD1d molecules. These results indicate that CD1d expression is intrinsic to prostate epithelium and suggest that prostate tumors can directly interact with iNKT cells.

Whether the CD1d expression on prostate tumor cells is functional and can activate iNKT cells was tested using the iNKT cell hybridoma DN32. We used TRAMP-C2 cells pulsed with the exogenous CD1d ligand α-GalCer to stimulate DN32 cells, which secrete only IL-2 upon TCR ligation [35]. In the absence of α-GalCer, TRAMP-C2 cells were not able to stimulate the DN32 cells, as seen by lack of IL-2 production (Fig. 3D, top bar). In contrast, DN32 responded vigorously to TRAMP-C2 cells in the presence of α-GalCer, which was specifically reduced by anti-CD1d mAbs.

### Murine prostate tumors defectively activate iNKT cells

**Figure 4:**
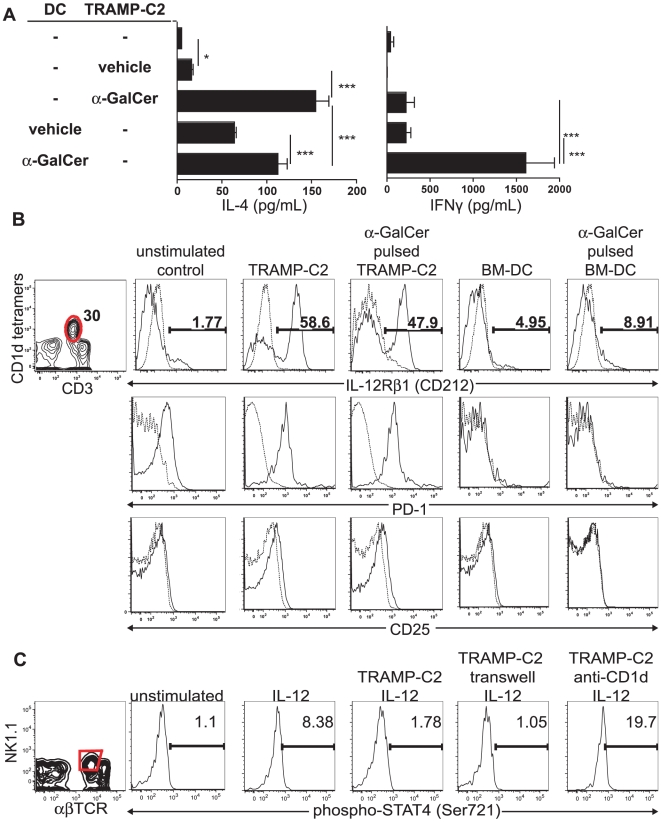
TRAMP-C2 cells partially activate iNKT cells by up-regulating activation markers and cell-contact dependently block STAT4 phosphorylation in iNKT cells. **A.** TRAMP-C2 and BM-DC were pulsed with α-GalCer or vehicle and used as stimulators for liver MNC as responder cells. Cell culture supernatants were measured 24 hrs (IL-4) and 48 hrs thereafter (IFNγ) by ELISA. **B.** WT hepatic MNC were exposed overnight to TRAMP-C2 cells, BM-DC, or left alone. Non-adherent cells were stained with indicated mAbs and analyzed by FACS. Histograms show expression of gated iNKT cells. **C.** Liver MNC were cultured overnight alone or in the presence of TRAMP-C2 cells, pretreated with saturating amounts of anti-mouse CD1d blocking mAbs (3C11) or separated by 0.4 µm transwell membranes. Cells were then stimulated for 30 mins. with 10 ng/ml rIL-12, immediately fixed and stained with mAbs against αβTCR and NK1.1. Cells were then permeabilized using methanol and intracellularly stained against phospho-STAT4 (Ser721). Histograms show phospho-STAT4 staining of electronically gated NKT cells (αβTCR^ +^NK1.1^+^) shown in left plot. Data shown are representative results of two experiments.

Having demonstrated the functionality of CD1d expression on TRAMP-C2 cells (Fig. 3D), we next asked which cytokine profile these tumor cells stimulate in primary iNKT cells. We exposed freshly isolated murine hepatic iNKT cells from healthy mice to TRAMP-C2 cells alone, and examined their cytokine production. BM-DC were used as positive controls for iNKT cell stimulation. As expected, iNKT cells produced high levels of both IL-4 and IFNγ in response to α-GalCer-pulsed DC, and IFNγ production was dependent on α-GalCer (Fig. 4A). However, iNKT cells produced IL-4, but no IFNγ, in response to TRAMP-C2. Pulsing TRAMP-C2 cells with α-GalCer increased the IL-4 expression by iNKT cells to a magnitude comparable of BM-DC (Fig. 4A, bar 3). This regimen, however, induced only marginal amounts of IFNγ from NKT cells.

These data prompted us to examine the of typical activation markers on iNKT cells upon stimulation with TRAMP-C2 cells. IL-12 mediates its effects by binding to a heterodimeric receptor comprised of two chains, of which the β1 chain is up-regulated during activation of conventional T cells and expressed at low levels on resting iNKT cells [36], [37]. As shown in Fig. 4B, we detected up-regulation of the IL-12Rβ1 chain 36 hrs after contact with TRAMP-C2 cells. Importantly, exposure to TRAMP-C2 cells without addition of α-GalCer was sufficient to achieve this effect. In contrast, DC, confirmed as superior iNKT cell stimulators (Fig. 4A), led only to a small increase in IL-12Rβ1 expression on iNKT cells, whether or not in the presence of α-GalCer (Fig. 4B).

Concomitantly, exposure to TRAMP-C2 cells (with and without α-GalCer) increased expression of PD-1 on iNKT cells, whereas BM-DC failed to induce this effect. iNKT cells express PD-1 upon activation *in vivo* and can subsequently enter an anergic-like state [38], [39]. How PD-1 is regulated *in vitro* is unclear. Despite this clear PD-1 upregulation, the IFNγ expression of iNKT cells stimulated with TRAMP-C2 cells was independent of interactions between PD-1 and PD-L1, which can be explained by the lack of PD-L1 expression by TRAMP-C2 cells (Figure S1). As expected, based on responses to IL-12, IL-12Rβ1 was induced by α-GalCer pulsed TRAMP-C2 cells (Fig. 4B). Interestingly, IL-12Rβ1 was also induced by TRAMP-C2 cells without α-GalCer (Fig. 4B).

Phosphorylation of STAT4 transcription factor in position Ser721 and Tyr693 is critical for IL-12R mediated signaling [40]. We tested whether preincubation of iNKT cells with TRAMP-C2 cells would block subsequent IL-12-mediated IL-12R signaling. Liver MNC of healthy WT mice were co-cultured with TRAMP-C2 cells and subsequently stimulated with IL-12 before STAT4 phosphorylation was examined by flow cytometry (Fig. 4C). IL-12 stimulation for 30 mins. resulted in the phosphorylation of STAT4 compared to un-stimulated control (MFI values 281 vs. 427). The pre-incubation with TRAMP-C2 cells blocked the STAT4 phosphorylation in iNKT cells (MFI 264). Furthermore, we tested whether this phosphorylation blockade was mediated by expression of CD1d on tumor cells or soluble mediators and co-cultured iNKT cells with TRAMP-C2 in the presence of CD1d blocking antibodies or separated by transwell membranes. Blocking of CD1d on TRAMP-C2 cells using saturating amounts before and during culture did not restore subsequent IL-12 mediated STAT4 phosphorylation (MFI 268). However, membrane separation between TRAMP-C2 and iNKT cells was sufficient to restore IL-12R signaling in iNKT cells, leading us to conclude that TRAMP-C2 cells CD1d-independently but cell-cell contact dependently block IL-12R signaling in iNKT cells.

### IL-12 and α-GalCer restore iNKT cell activation in response to prostate tumor cells

**Figure 5:**
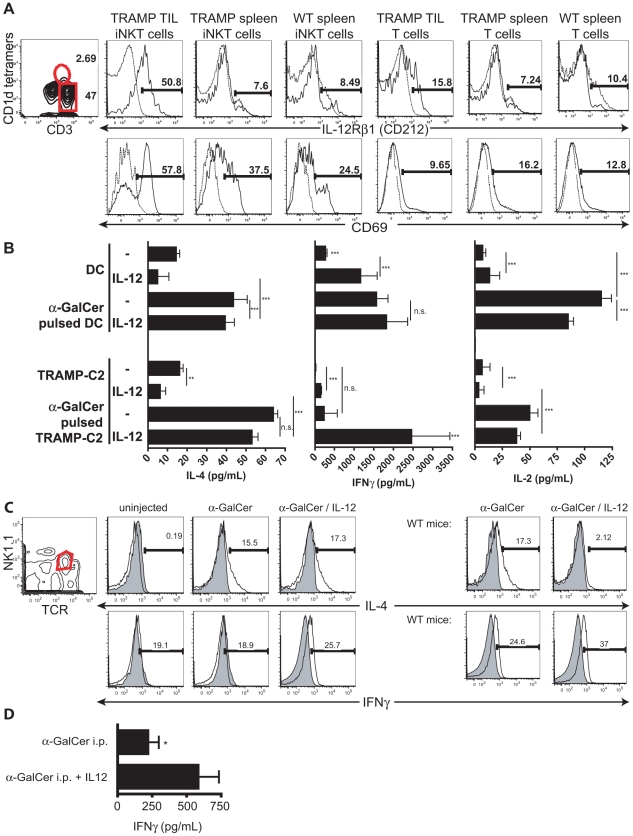
IL-12 combined with α-GalCer restores the ability to activate iNKT cells in response to prostate tumor cells. **A.** IL12Rβ1 expression on TIL-iNKT and T cells from primary TRAMP prostate tumors, TRAMP spleen and WT spleen *ex vivo*. Contour plot shows presence of iNKT (CD3^+^CD1d tetramer^+^) and T cells (CD3^+^CD1d tetramer^−^) from TRAMP prostate tumors, gated on CD45^+^ TIL. Data shown are representative of 2 experiments. **B.** Liver MNC were cultured in the presence or absence of IL-12 with TRAMP-C2 or DC pulsed with α-GalCer or vehicle for 24 or 48 hrs. Cell culture supernatants were tested for cytokine secretion by ELISA. Data shown are representative of 3 experiments (*, P<0.05; ***, p<0.001; n.s., not significant). **C.** WT C57BL/6 mice were injected with TRAMP-C2 cells s.c. or left untreated. Two weeks thereafter, mice were injected with 150 µg Brefeldin-A and 1 µg α-GalCer with or without IL-12. Five hours later, spleen cells were stained with mAbs against αβTCR and NK1.1, followed by permeablization and subsequent intracellular staining with mAbs against IL-4, IFNγ, or rat IgG isotype controls. Histograms show total cytokine expression of gated NK1.1^+^ cells. **D.** Tumor-bearing TRAMP mice were i.p. injected with α-GalCer with or without recombinant IL-12. IFNγ levels from sera taken 90 mins later were determined by ELISA (n = 3, *, p<0.05).

In light of the IL-12R up-regulation observed in vitro (Fig. 4B), we further characterized TIL-iNKT cells present in TRAMP tumors. Although we did not see any up-regulation of PD-1 or CD25 molecules on iNKT cells in TRAMP mice (data not shown), we consistently found strong up-regulation of IL-12Rβ1 on TIL-iNKT cells in tumor, but not from spleens, in both groups (Fig. 5A). The up-regulation of IL-12Rβ1 was specific for iNKT cells, as it was not detectable on conventional T cells (Fig. 5A), further corroborating our hypothesis of CD1d-mediated iNKT cell partial activation. Additionally, iNKT cells present in TRAMP tumors specifically expressed higher levels of the early activation marker CD69 as compared to iNKT cells in spleens of both groups and conventional T cells in all groups (Fig. 5A).

It has been well documented that IL-12 is an adjuvant for the IFNγ production by iNKT cells [41], [42]. The observed up-regulation of the IL-12R led us to determine whether addition of IL-12 induces IFNγ production of iNKT cells in response to TRAMP-C2 cells. Freshly isolated iNKT cells from healthy mice were co-cultured in the presence of TRAMP-C2 cells or BM-DC with addition of IL-12. Similar to results seen in Fig. 4A, in the absence of α-GalCer, DC stimulated modest production of IL-4, but not IFNγ, from iNKT cells (Fig. 5, bar 1). The addition of IL-12 enhanced IFNγ production of iNKT cells (Fig. 5B, bar 2), as previously described [42]. Furthermore, α α-GalCer pulsed DC induced secretion of IL-2, IL-4, and IFNγ by iNKT cells.

Corroborating with the results shown in Fig. 4, TRAMP-C2 cells without addition of α-GalCer stimulated IL-4 production in iNKT cells, but were not sufficient to induce IFNγ responses (Fig. 5A, bar 5). Furthermore, TRAMP-C2 cells did not induce IL-2 responses by iNKT cells. Upon pulsing with α-GalCer, TRAMP-C2 cells induced a significantly higher IL-2 and IL-4 production, but only minimal amounts of IFNγ in iNKT cells (Fig. 5B, bar 7).

When adding IL-12 to stimulations of iNKT cells by TRAMP-C2 cells, we observed significant albeit minimal levels of IFNγ produced by iNKT cells (Fig. 5B, bar 6). The addition of IL-12 to α-GalCer-pulsed TRAMP-C2 as stimulators was sufficient to induce a significant production of IFNγ in iNKT cells. These data were corroborated by intracellular cytokine staining performed on in vivo activated NK1.1^+^ cells isolated from hosts of subcutaneous TRAMP-C2 tumors. TRAMP-C2 cells were s.c. injected into WT C57BL/6 mice. Two weeks after tumor seeding iNKT cells were activated by i.p. injections of 1 µg α-GalCer with or without 500 ng IL-12. To inhibit cytokine export from cells, mice received injections of Brefeldin-A five hours before liver MNC (Fig. 5C) and spleen cells (data not shown) were isolated and stained for flow cytometry. Injection of α-GalCer into untreated WT mice stimulated iNKT cells to production of both IFNγ and IL-4. Comparable to in vitro stimulation data (Fig. 5B), iNKT cells from liver and spleen of TRAMP-C2 injected animals produced IL-4 but not IFNγ in response to α-GalCer (Fig. 5C; not shown). The simultaneous injection of IL-12 and α-GalCer did restore the ability to produce IFNγ. Comparable data were obtained after injecting tumor-bearing TRAMP mice with α-GalCer with and without the addition of recombinant IL-12 showing a significant increase of IFNγ serum levels in response to α-GalCer and IL-12 compared to α-GalCer alone (Fig. 5D).

These data show that TRAMP-C2 cells stimulate iNKT cells to produce IL-4 but not IFNγ, which could not be stimulated by addition of α-GalCer. The addition of both high-affinity CD1d agonist α-GalCer and the adjuvant IL-12 were required to induce IFNγ production in iNKT cells in vitro and in vivo. IL-12 injection concomitant to α-GalCer stimulation reversed the systemic iNKT defects TRAMP-C2 cells induced and restored the activation of iNKT cells.

**Figure 6:**
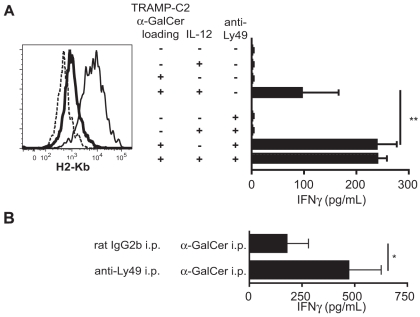
Ly49 blockade concomitant with α-GalCer restores IFNγ production in iNKT cells. **A.** Liver MNC were cultured with α-GalCer loaded or unloaded TRAMP-C2 cells in the presence or absence of exogenous IL-12 and upon blocking with Ly49C/F/H/I molecules on iNKT cells as indicated. Cell culture supernatants were tested for cytokine secretion by ELISA. Histogram shows MHC- (H2-Kb) expression on TRAMP-C2 cells (solid line) or WT C57Bl/6 DC (bold line), isotype controls (dashed line). **B.** Tumor-bearing TRAMP mice were i.p. injected with each 50 uG anti-Ly49C/F/H/I mAbs 18 h and 2 hours before 2 µG α-GalCer was injected i.p. Graphs shows IFNγ serum levels (n = 3, p<0.05).

iNKT cells express both activating and inhibitory NK-like receptors including Ly49 receptor types recognizing MHC class I molecules. Subtypes C, I, and F of the Ly49 receptor contain ITIM motif in the cytoplasmic domain and thereby suppress positive signals exerted by cytokine and other receptors [43]. MHC class I staining of TRAMP-C2 cells revealed the expression of MHC class I on the surface (Fig. 6, histogram) which is supported with earlier of TRAMP-C2 cells as MHC class I low expressing cells [44]. To address a conceivable negative effect of inhibitory Ly49 receptors expressed on iNKT cells, Ly49 expression on iNKT cells was blocked. iNKT cells were then stimulated with α-GalCer pulsed (or untreated) TRAMP-C2 cells in the presence or absence of IL-12 and IFNγ secretion measured by ELISA. Confirming data shown in Fig. 5 both α-GalCer and were required for IFNγ production in iNKT cells stimulated by TRAMP-C2. Blockade of Ly49 expression on iNKT cells during cocultures with untreated TRAMP-C2 cells did not elicit any detectable IFNγ production (Fig. 6, bar graph). Blockade of Ly49C/F/I/H receptors on liver MNC during co-incubation with TRAMP-C2 cells in the presence of exogenous IL-12 restored IFNγ secretion to levels comparable those observed after co-incubation with α-GalCer loaded TRAMP-C2 cells and IL-12.

To confirm a role of Ly49 receptors *in vivo*, we injected TRAMP mice with Ly49 antibodies before iNKT cells were activated by α-GalCer administration. Comparable to *in vitro* results (Fig. 6, upper graph), Ly49 blockade significantly increased IFNγ serum levels elicited by α-GalCer administration. This suggests a role for NK-like inhibitory receptors in iNKT cell defects. Comparable IFNγ levels were also induced by simultaneous injection of α-GalCer and IL-12 (Fig. 5D).

Collectively, these data indicate that murine prostate tumor cells induce a partial activation state in iNKT cells characterized by STAT4 phosphorylation blockade. Although iNKT cells in this state up-regulate the IL-12R, they are unable to respond to IL-12 in the absence of high affinity ligand. iNKT cell defects were apparently at least partially mediated by MHC-I/Ly49 interaction between prostate tumor and iNKT cells.

## Discussion

Systemic numerical and functional iNKT cell defects have been observed in cancer patients. iNKT cells of cancer patients showed a significant reduction of IFNγ in response to TCR-mediated activation *in vitro* [5]–[9]. The mechanism(s) underlying the resulting iNKT cell defects remain obscure. We sought to develop a suitable mouse model to investigate such. In the TRAMP mouse prostate tumor model, we found iNKT cells from cancer-bearing mice exhibit functional defects similar to those observed in humans. Specifically, upon activation with the CD1d ligand α-GalCer, iNKT cells from tumor-bearing mice exhibited a diminished production of both Th2 cytokines and IFNγ. In addition, DCs from these mice did not produce IL-12 upon interaction with iNKT cells. These data are supported by a recent study of Bellone et al. who reported a defective production of IFNγ and IL-4 of TRAMP mice in response to α-GalCer [5].

We characterized the interaction of iNKT cells from TRAMP mice *ex vivo* compared to iNKT cells from healthy mice with the murine tumor cell line TRAMP-C2. TRAMP-C2 cells, human CaP lines and primary TRAMP tumors expressed CD1d. TRAMP-C2 cells activated iNKT cells from healthy mice in a CD1d-dependent fashion to produce IL-4, but not IFNγ. These findings prompted us to hypothesize that these tumor cells may aberrantly activate iNKT cells. TRAMP-C2 cells induced the expression of the activation markers CD25, PD-1 and IL-12Rβ1 on iNKT cells. It should be stressed that TRAMP-C2 tumor cells activated iNKT cells without addition of the exogenous ligand α-GalCer. This is corroborated by our findings that TRAMP-C2 cells induced the secretion of IL-4, but not IFNγ. Production of IL-4 has been shown to occur independently of co-stimulatory molecules presented by CD1d^+^ DC, whereas the production of IFNγ is enhanced by IL-12 produced by DC [15]. Importantly, while iNKT cells up-regulated the IL-12R in response to TRAMP-C2 cells, the addition of IL-12 alone was not sufficient to stimulate their IFNγ production. Additionally, TRAMP-C2 cells blocked STAT4 signaling downstream of the IL-12 receptor. Phosphorylation in residues Ser721 and Tyr693 is critical for transactivation of STAT4 [40]. This inhibition was cell-cell contact dependent. The lack of effect of saturating amounts of blocking CD1d antibody strongly suggests IL-12R inhibition is independent of CD1d expression on TRAMP-C2 cells. This is corroborated by the inhibitory effect of Ly49 on NKT cells during culture with α-GalCer loaded TRAMP-C2 cells and in tumor-bearing TRAMP mice *in vivo*.

The functional inhibition of IFNγ production was reversible by the simultaneous addition of both IL-12 and the high-affinity ligand α-GalCer, thus showing that tumor cells reversibly inhibit iNKT cells. This inhibition can be overcome by addition of strong TCR signals, such as provided by the high affinity ligand α-GalCer (Fig. 6). Our further results suggest inhibitory Ly49 receptors play a role in the observed iNKT cells defects. TRAMP-C2 cells express MHC class I molecules at low levels, thus providing a potential explanation for our findings that Ly49 blockade alone does not completely rescue the IFNγ production, but still requires the presence of α-GalCer as a high-affinity ligand for CD1d. In the absence of α-GalCer TRAMP-C2 were able to stimulate the production of IL-2 in DN32 and IL-4 in iNKT cells. Further studies will be needed to elucidate the identity of the CD1d ligand expressed by TRAMP-C2 cells.

Additionally, we show that subcutaneously injected TRAMP-C2 cells inhibit iNKT cells systemically to i.p. injection of α-GalCer, which was reversible by simultaneous administration of α-GalCer and IL-12. Similar effects were obtained by blockade of inhibitory Ly49 receptors in TRAMP mice. Since free injected α-GalCer injected *in vivo* binds DCs and other APC, we conclude that TRAMP-C2 tumors systemically inhibit the response of iNKT cells to α-GalCer mediated activation by other cells. The lack of cell-contact between subcutaneous TRAMP-C2 and splenic iNKT cells suggests this inhibition is mediated by soluble factors. Systemic APC defects in iNKT cell presentation in cancer patients and rodent tumor models have been reported [45].

**Figure 7:**
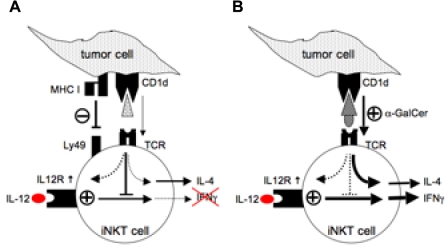
Proposed working model of tumor cell-induced functional iNKT cell defects in TRAMP prostate cancer. **A.** Tumor cells deliver 2 signals to iNKT cells: CD1d:TCR-mediated signal is sufficient for a partial activation, i.e. the up-regulation of activation markers and secretion of IL-4 by iNKT cells. MHC-I:Ly49 interaction inhibits enhancement of IFNγ production by iNKT cells. **B.** Stimulation with high-affinity ligand α-GalCer and IL-12 bypasses tumor cell-induced block of IFNγ production.

We conclude from these findings that tumor cells directly block the production of IFNγ cytokines in iNKT cells. iNKT cells may store cytokine mRNA, allowing them to respond rapidly upon TCR-mediated activation [46]. Thus, it is conceivable that prostate tumor cells, in addition to CD1d-mediated signals activating iNKT cells deliver additional signals that block the translation or export of IFNγ in iNKT (Fig. 7). Whether the IFNγ blockade is mediated by tumor-derived soluble factors is currently not known.

These *in vitro* findings are further corroborated by our *ex vivo* data showing the expression IL-12Rβ1 and CD69 on iNKT cells in TRAMP prostate tumors. Despite the high expression of CD69, TIL-iNKT cells did not express CD25, which upon activation is expressed at later timepoints compared to CD69. Whether this is due to a higher turnover of TIL-iNKT cells in the tumor by apoptosis, remains to be clarified. Future experiments will be needed to investigate whether the aberrant activation of iNKT cells seen in murine prostate tumors can be observed in advanced prostate cancer patients Notably, like human prostate tumors and CaP cell lines [25], [26], TRAMP prostate tumor cells secrete high amounts of the chemokine CCL2 and attracted iNKT cells into the TME without detectable depletion of iNKT cells elsewhere. This argues for a higher turn-over rate. Specifically, only iNKT cells in the prostate tumors but not spleens of tumor-bearing mice (or control mice) showed an activated phenotype. This suggests that the local presence of tumor cells is required for this effect.

In conclusion, by using the T-antigen induced prostate cancer TRAMP mouse, whose disease resembled features of human PCa disease, we developed a suitable model to study iNKT cell defects and their reversion. We show that prostate tumor cells, induce a novel and partial activation state in iNKT cells. Concomitantly, prostate tumor cells directly inhibit secretion of IFNγ as a effector function in iNKT cells. This effect was resistant to IL-12. The correction of these iNKT cell defects by CD1d ligand α-GalCer with IL-12 might support future development of IL-12R and iNKT-targeted therapeutic approaches.

## Supporting Information

Figure S1 PD-L1 blockade does not restore TRAMP-C2 mediated IFNγ secretion in iNKT cells. TRAMP-C2 cells were loaded with α-GalCer vigorously washed and incubated with anti-PDL1 mAbs or isotype controls before freshly isolated liver MNC prestained with mAbs against αβTCr and NK1.1 were added as responder cells. Cocultures were carried out in the presence of Brefeldin A for 6 hours before intracellular IFNγ staining was performed. Data shown are of iNKT cells gated as αβTCR+NK1.1+ cells. Results shown are representative of two experiments. Bottom right histogram: TRAMP-C2 cells do not express PD-L1. TRAMP-C2 cells (bold line) or BM-DC (dashed line) were stained with anti-PD-L1 TRAMP-C2or rat IgG2b isotype control mAbs (dotted line), followed by anti-rat IgG FITC mAbs and analyzed by FACS.
(1.32 MB EPS)
